# Cooperative Game-Based Energy Efficiency Management over Ultra-Dense Wireless Cellular Networks

**DOI:** 10.3390/s16091475

**Published:** 2016-09-13

**Authors:** Ming Li, Pengpeng Chen, Shouwan Gao

**Affiliations:** 1 School of Computer Science and Technology, China University of Mining and Technology, Xuzhou 221116, China; chenp@cumt.edu.cn (P.C.); gaoshouwan@cumt.edu.cn (S.G.); 2 Key Laboratory of Gas and Fire Control for Coal Mines, China University of Mining and Technology, Xuzhou 221116, China

**Keywords:** ultra-dense wireless cellular networks, cooperative game, energy efficiency, power coordination

## Abstract

Ultra-dense wireless cellular networks have been envisioned as a promising technique for handling the explosive increase of wireless traffic volume. With the extensive deployment of small cells in wireless cellular networks, the network spectral efficiency (SE) is improved with the use of limited frequency. However, the mutual inter-tier and intra-tier interference between or among small cells and macro cells becomes serious. On the other hand, more chances for potential cooperation among different cells are introduced. Energy efficiency (EE) has become one of the most important problems for future wireless networks. This paper proposes a cooperative bargaining game-based method for comprehensive EE management in an ultra-dense wireless cellular network, which highlights the complicated interference influence on energy-saving challenges and the power-coordination process among small cells and macro cells. Especially, a unified EE utility with the consideration of the interference mitigation is proposed to jointly address the SE, the deployment efficiency (DE), and the EE. In particular, closed-form power-coordination solutions for the optimal EE are derived to show the convergence property of the algorithm. Moreover, a simplified algorithm is presented to reduce the complexity of the signaling overhead, which is significant for ultra-dense small cells. Finally, numerical simulations are provided to illustrate the efficiency of the proposed cooperative bargaining game-based and simplified schemes.

## 1. Introduction

With the growth of wireless cellular communication over the past decade, smart mobile devices, such as smart phones, tablets, and laptops, are being rapidly developed and widely used for bandwidth-hungry mobile Internet applications [1,2,3,4,5]. There is a broad consensus in the wireless industry that this trend will continue for several years. To support the exponential increase of wireless data traffic, the capacity of wireless cellular networks needs to grow accordingly. The wireless industry has taken on the challenge of cost-effectively supporting a 1000 × increase in the wireless capacity demand over the next decade, which is becoming a hot research topic in both industries and academics [6]. 

In light of the observation by Martin Cooper—the pioneer of wireless communications—wireless system capacity can be increased by increasing the wireless nodes, expanding the radio spectrum, and improving the link efficiency [1,2]. In recent research, millimeter-wave communication was proposed for expanding the system bandwidth and increasing the system capacity [4]. Furthermore, a massive multiple-input multi-output technology [3] was presented to improve the spectrum efficiency of 5G wireless cellular communication systems. The small-cell conception has been found to increase the throughput and reduce the energy consumption in wireless scenarios [5]. These ingredients for wireless-capacity enhancement may be regarded as having the purpose of “network densification” [1]. 

To realize seamless coverage for future wireless cellular networks, a larger number of small cells must be densely deployed, forming ultradense wireless cellular networks. Initial studies involving ultradense wireless cellular networks are explored in [1,6,7,8,9]. Bhushan et al. discussed challenges and opportunities in network densification, which are regarded as the key technology for wireless evolution in the next decade, and analyzed the spatial densification situation and spectrum aggregation situation, i.e., small-cell dense deployment and radio spectrum utilization in diverse bands for 5G networks [1]. On the basis of WiFi and long-term evolution (LTE) technologies, a joint intra-cell and inter-cell resource-allocation coordination scheme is proposed [7]. Soret et al. presented two interference coordination algorithms for the time and frequency domains for LTE-A dense wireless networks [8]. Ge et al. proposed a distributed gateway network architecture for 5G ultra-dense wireless cellular networks, and investigated the backhaul capacity and the backhaul energy efficiency (EE) [6]. The spectral efficiency (SE) and EE of ultra-dense wireless cellular networks under different deployment strategies were investigated in [9]. 

Future wireless cellular networks should be more environmentally friendly [10,11]. Hence, the corresponding structural design, operation, and implementation should pursue minimal impacts on the human environment. As observed in [12], energy-efficient wireless communication has recently drawn increasing attention from the research community, especially with the explosive increase of high-data-rate applications. There are adequate energy-efficiency metrics of primary importance, which are directly related to optimization schemes across all the protocol layers. The most popular one is “bits-per-Joule”, which is defined as the total throughput for unit energy consumption. According to this metric, in [13], EE analysis at the link level is provided, where the transmission power is considered as the primary constraint. In [14], the bits-per-Joule metric at the network level is analyzed, and the number of nodes in the network is proven to be an important factor to increase the EE. Other metrics, such as the SE (Hz/J) and deployment efficiency (DE, bits/€), aim to observe the achieved utility of the different resources. For example, the DE focuses on the financial aspects of the system efficiency [15], which is suitable for a cellular operator concerned about revenue earnings. This metric contains a careful treatment of the capital expenditure (CapEx) and operational expenditure (OpEx) of the wireless network. For a core network, which cares more about the capacity, the SE is more suitable for explaining the relationship between the capacity and the energy consumption [16].

In ultra-dense wireless cellular networks, the small cell is a general term adopted in LTE standards, which refer to picocells and femtocells in a coverage radius of 10–300 m [17]. Compared with orthogonal deployment, spectrum sharing deployment between multiple small cells and macro cells is more attractive owing to its easier implementation and more efficient utilization of the frequency band [18]. As a result, the network SE is improved with frequency reuse and spectrum sharing. At the same time, with the coexistence of multiple small-cell base stations (SeNBs) and macro-cell base stations (MeNBs), the path loss between a user and its base station is reduced, which increases both the signal power and the inter-tier interference, and effectively dwarfs the effect of thermal noise. Thus, interference mitigation is essential for network efficiency management in ultradense wireless cellular networks, which requires adaptive resource coordination among the whole network. 

According to [19,20], interference-aware power coordination among SeNBs and MeNBs is a critical issue for mitigating both inter-tier and intra-tier interference and achieving good SE. The conventional power coordination schemes use a central controller to realize convex optimization by a complex signaling overhead, which is not suitable for a distributed ultra-dense wireless cellular network. Game theory provides a natural model for handling multiple interactively interfering entities to seek a solution for maximizing the utility of every entity [21,22]. Many studies on power coordination and SE optimization have adopted a non-cooperation Nash game-theoretic approach. In [23], the non-cooperative power allocation considering the signal-to-interference-plus-noise-ratio (SINR) is proposed to relieve the interference from femtocells to macro cells. In [24], a power control scheme based on the Stackelberg game is formulated to maximize the capacity of femtocells under an inter-tier interference constraint. A non-cooperative power and subchannel resource allocation scheme for co-channel femtocells is presented with transmitting protection for macro-cell users [25].

Although the aforementioned power-coordination strategies based on the strategic non-cooperative Nash game could simply reflect the hierarchical relationship between MeNBs and SeNBs, they are unable to capture the inherent coordination actions among different base stations. Moreover, players in a non-cooperative game act rationally and selfishly to maximize their individual utility [26], which might introduce additional interference to other cells. The strategies fail to guarantee fairness among different small cells, where the Nash equilibrium is not always efficient. To improve the Pareto-optimality of the non-cooperative game, schemes have been introduced such as pricing of the interference [27], the virtual referee [28], uniform and differential pricing game of resource scheduling [29], and repeated game [30]. However, backhauls are necessary for massive information exchange or aggregation to achieve accurate pricing. Furthermore, pricing scheme may lead to a slow convergent or even divergent power-control algorithm. On the other hand, the cooperative game model [31,32] is more suitable for resource allocation in ultra-dense wireless cellular networks, which has been proven in the economic field. A cooperative bargaining game was demonstrated to achieve the optimal social solution by maximizing a Nash-product utility function [33].

Moreover, game theory-based energy-efficient resource allocation has been studied for wireless networks [11,32,34]. In [34], the Nash equilibrium of a power-control game was proposed to enhance the EE. In [11], the EE is investigated as a significant requirement of future HetNets, especially when SeNBs and MeNBs are densely deployed. The work in [35] addressed the fundamental tradeoff between the EE and SE in downlink OFDMA networks. The work in [32] explored the tradeoff between traffic offloading from the MeNB and the energy consumption of SeNBs in a cognitive small-cell network.

According to the aforementioned literature, although ultradense wireless cellular networks have good SE performance, the serious interference must be reduced by potential cooperation schemes, especially in the comprehensive consideration of the network EE. Power coordination plays an important role in enhancing both the SE and EE, but few studies have focused on the DE and the inherent interaction between these three metrics (SE, EE and DE) under one unified cooperative game model in ultra-dense wireless cellular networks.

Herein, we propose a cooperative bargaining game-based power-coordination scheme for ultra-dense wireless cellular networks to improve the comprehensive EE with consideration of the inter-tier and intra-tier interference limitations and maximum power constraints. The main contributions are summarized as follows:

- We formulate the power-coordination problem in macro cells and small cells as a cooperative Nash bargaining game to improve the comprehensive EE, where the inter-tier and intra-tier interference limitations are imposed to provide reliable transmission for the network users, and the maximum power is considered to guarantee the fairness for the base stations in each cell.
- We define a unified energy-efficient utility function for the optimization problem in ultradense wireless cellular networks, where the near optimal cooperative bargaining power-coordination strategy is derived by introducing time-sharing variables and the Lagrangian function. Accordingly, the closed-form power-coordination solutions with consideration of both interference mitigation and energy saving are derived, which show the convergence of a Pareto-optimal equilibrium for the cooperative game.
- A simplified algorithm is proposed to combat the complicate signaling overhead, which is a challenge in the scenario of the ultradense deployment of small cells, and yields a suboptimal power-coordination solution.
- The proposed algorithm is evaluated by numerical simulations, which illustrates the convergence property and the efficiency of our power-coordination scheme, allowing a good tradeoff between three different energy-efficiency metrics.

The remainder of the paper is organized as follows: Section 2 introduces the system model and the energy-efficiency model. Section 3 provides the basics for the Nash cooperative bargaining game and a unified energy-efficient utility function. Section 4 provides the closed-form solution, the algorithm implementation, and a simplified algorithm in ultra-dense wireless cellular networks, and in Section 5, the performances of the proposed algorithms are evaluated by simulations. Finally, we conclude the work in Section 6.

## 2. System Model and Network Energy-Efficiency Model

### 2.1. System Model

**Figure 1:**
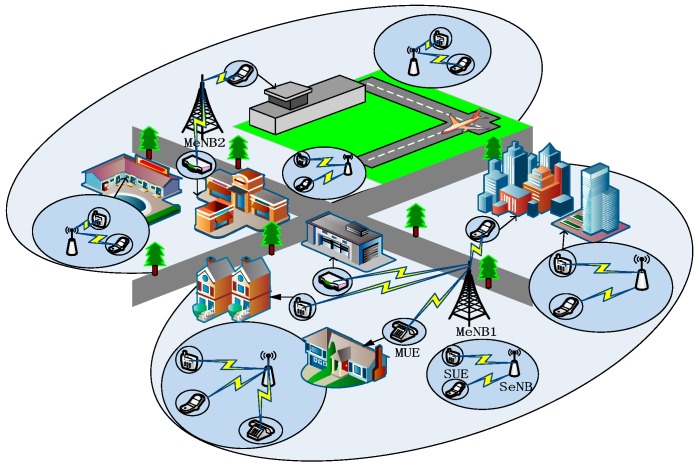
Ultradense deployment scenario of a wireless cellular network.

As previously mentioned, the ultradense deployment of low-power nodes is an important method for increasing the system capacity. Thus, we consider a two-tier heterogeneous network, as illustrated in Figure 1, where multiple co-channel low-power nodes are overlaid on an existing macrocell controlled by a MeNB. We assume that both tiers share the spectrum. As shown in Figure 1, many SeNBs dominate their respective small cells. With the increase of SeNBs, inter- and intra-tier interference seriously affects and restricts the network performance. For example, the macrocell user equipment (MUE) suffers significant performance degradation owing to the nearest SeNB. Therefore, it is crucial to research the interference influence on network EE.

Here, we focus on the resource allocation in the downlink of this network. Let M = {1, 2, 3, …, *M*} represent the MeNB set. Every macrocell is covered with *N* SeNBs, and N = {1, 2, 3, …, *N*} represents the SeNB set. In each macro-cell, there are *I* MUEs and *J* small-cell user equipments (SUEs) associated with each SeNB. We set *g_m,i_* and *g_n,j_* as the channel power gains from MeNB and SeNB to their associated users MUE and SUE, respectively, where *m*∈{1, 2, …, *M*}, *i*∈{1, 2, …, *I*}, *n*∈{1, 2, …, *N*}, *j*∈{1, 2, …, *J*}; *p_m_* and *p_n_* are the transmission power for the MeNB_m_ and SeNB_n_, respectively, in the downlink of the macro-cell and the small-cell.

Thus, the received SINR of the *i*th MUE_i_ at the *m*th MeNB_m_ is given by:

(1) $$\gamma_{m,i}\;=\;\frac{p_{m}g_{m,i}}{\sum_{m\prime \neq m,m\prime =1}^{M}p_{m\prime }g_{m\prime ,\{m,i\}}+\sum_{n=1}^{N}p_{n}g_{n,\{m,i\}}+\sigma_{m,i}^{2}}$$

where *p_m_* is the transmission power of the other MeNBs besides MeNB_m_, *g_m′_*_,{*m,i*}_ is the channel gain on MUE_i_ from other MeNBs, *g_n_*_,{*m,i*}_ is the channel gain on MUE_i_ from SeNBs overlaid on the coverage of MeNB_m_, and $\sigma_{m,1}^{2}$ is the additive white Gaussian noise (AWGN) power. In the aforementioned equation, $\sum_{n=1}^{N}p_{n}g_{n,\left\{m,i\right\}}$ represents the inter-tier interference on MUE_i_, and $\sum_{m^{\prime }\neq m,m^{\prime }=1}^{M}p_{m\prime }g_{m\prime ,\left\{m,i\right\}}$ represents the intra-tier interference on MUE_i_.

The channel-to-interference-plus-noise ratio (CINR) of MUE_i_ is:

(2) $$h_{m,i}=\frac{g_{m,i}}{\sum_{m\prime \neq m,m\prime =1}^{M}p_{m\prime }g_{m\prime ,\{m,i\}}+\sum_{n=1}^{N}p_{n}g_{n,\{m,i\}}+\sigma_{m,i}^{2}}$$

Hence, Equation (1) can be simplified as:

(3) $$\gamma_{m,i}=p_{m}h_{m,i}$$

Similarly, the received SINR of the *j*th SUE_j_ at the *n*th SeNB_n_ is given by:

(4) $$\gamma_{n,j}=\frac{p_{n}g_{n,j}}{\sum_{n\prime \neq n,n\prime =1}^{N}p_{n\prime }g_{n\prime ,\{n,j\}}+\sum_{m=1}^{M}p_{m}g_{m,\{n,j\}}+\sigma_{n,j}^{2}}$$

where *p_n′_* is the transmission power of the other SeNBs besides SeNB_n_, *g_n′_*_,{*n,j*}_ is the channel gain on SUEj from other SeNBs, *g_m_*_,{*n,j*}_ is the channel gain on SUE_j_ from the MeNBs around SeNB_n_, and $\sigma_{n,j}^{2}$ is the AWGN power. $\sum_{n=1}^{M}p_{m}g_{m,\left\{n,j\right\}}$ represents the inter-tier interference on SUE_j_, and $\sum_{n^{\prime }\neq n,n^{\prime }=1}^{N}p_{n\prime }g_{n\prime ,\left\{n,j\right\}}\;$represents the intra-tier interference on SUE_j_. We assume that *h_n,j_* is the CINR of SUE_j_. Then, Equation (4) can be simplified as:

(5) $$\gamma_{n,j}=p_{n}h_{n,j}$$

According to Shannon’s capacity formula, the achievable capacities of MUE_i_ and SUE_j_ are:

(6) $$C_{m,i}=\log_{2}(1+p_{m}h_{m,i})$$

(7) $$C_{n,j}=\log_{2}(1+p_{n}h_{n,j})$$

### 2.2. Network Energy-Efficiency Model

In this paper, we aim to maximize the total EE of the whole network. An appropriate energy-efficiency metric is most important, which is directly related to the optimized strategies across all the layers. Here, a network energy-efficiency model is defined with regard to three aspects—spectrum, energy, and deployment—to realize comprehensive energy-efficient management.

The SE is defined as the network throughput for unit bandwidth, and is a widely accepted metric for wireless-network optimization. The downlink throughput is related to the transmission power of the base station in the network, which includes MeNBs and SeNBs. Therefore, we analyze the SE of SeNBs and MeNBs separately. According to Equations (6) and (7), the spectral efficiencies of MeNB_m_ and SeNB_n_ can be expressed as:

(8) $$SE_{m}=\sum_{i=1}^{I}\log_{2}(1+p_{m}h_{m,i})$$

(9) $$SE_{n}=\sum_{j=1}^{J}\log_{2}(1+p_{n}h_{n,j})$$

where $SE_{m}$ and $SE_{n}$ are calculated as the sum capacity of all the MUEs and SUEs associated with the *m*th MeNB_m_ and the *n*th SeNB_n_.

The EE is defined as the network throughput per unit energy consumption and is mostly considered during network operation. According to [10], a large electricity bill results from the large energy consumption of a wireless base station. Thus, we analyze the EE of each base station, which reflects the EE of the whole network. According to [15], power consumption includes two parts: transmission power consumption and circuit power consumption. 

Therefore, the EE of MeNB_m_ and SeNB_n_ can be expressed as:

(10) $$EE_{m}=\frac{\sum_{i=1}^{I}\log_{2}(1+p_{m}h_{m,i})}{a_{sitem}*P_{m}+b_{sitem}}$$

(11) $$EE_{n}=\frac{\sum_{j=1}^{J}\log_{2}(1+p_{n}h_{n,j})}{a_{siten}*P_{n}+b_{siten}}$$

where a_sitem_*P_m_ represents the *m*th MeNB_m_ transmission power consumption; *b_sitem_* is the circuit power consumption; *a_sitem_* is the *m*th MeNB_m_ power-conversion efficiency, accounting for the power amplifier efficiency, feeder loss, extra loss in transmission-related cooling, etc. Similarly, a_siten_*P_n_ is the *n*th SeNB_n_ transmission power consumption, *b_siten_* is the circuit power consumption, and *a_siten_* is the *n*th SeNB_n_ power-conversion efficiency.

The DE is defined as the network throughput for unit deployment cost and is an important network-performance metric for users. According to [15], the deployment cost includes the CapEx and OpEx. Key cost drivers with respect to the CapEx and OpEx are summarized in Table 1. 

With the increase of base stations, the base station-related expenditure accounts for a large proportion. Thus, discussing the cost efficiency of each base station is important for the DE of the whole network. According to [15], the annual average cell deployment cost can be expressed as:

(12) $$\begin{array}{l} \overset{-}{C}(R)=C^{Ca}(R)/10+C^{Op}(R)\\ =\{\begin{array}{c} 0.86c_{0}+c_{1}{\overset{-}{E}}_{site}(R),R\geq 0.5\\ 0.57c_{0}+c_{1}{\overset{-}{E}}_{site}(R),0.1\leq R\leq 0.5 \end{array} \end{array}$$

where *R* is the cell radius, whose unit is km; $c_{0}$ denotes the equipment cost of a base station (unit: €); and $c_{1}$ denotes the electricity charge in units of K€/J.

Therefore, the DE of SeNB_n_ and MeNB_m_ can be expressed as:

(13) $$DE_{m}=\frac{\sum_{i=1}^{I}\log_{2}(1+p_{m}h_{m,i})}{c_{1m}*(a_{sitem}*P_{m}+b_{sitem})+c_{om}}$$

(14) $$DE_{n}=\frac{\sum_{j=1}^{J}\log_{2}(1+p_{n}h_{n,j})}{c_{1n}*(a_{siten}*P_{n}+b_{siten})+c_{0n}}$$

where *c*_1m_*(a_sitem_*P_m_ + *b_sitem_*) represents the *m*th MeNB_m_ OpEx; *c*_o*m*_ is the CapEx; and *c*_1*m*_ is the *m*th MeNB_m_ electricity charge in units of K€/W. Similarly, *c*_1n_*(a_siten_*P_n_ + *b_siten_*) represents the *n*th SeNB_n_ OpEx; *c*_o*n*_ is the CapEx; and *c*_1*n*_ is the *n*th SeNB_n_ electricity charge.

## 3. Cooperative Game in Ultra-Dense Wireless Cellular Networks

In this section, the basic definitions of bargaining solutions of cooperative game theory are briefly introduced. Then, the utility function is designed according to the bargaining games.

### 3.1. Definitions and Concepts of Cooperative Game

Let K = {1, 2,…, *K*} be the set of players, which are base stations in this paper. Let *S* be the energy-efficient strategy of the players, with *P_n_* being the power-allocation strategy space. Player *k* has the utility/payoff function *U_k_*, which can be derived from the allocated power. $U_{k}^{\min}$ is the minimum desired payoff that player *k* expects, which is called the disagreement point. In cooperative games, players attempt to reach an agreement that provides a mutual advantage. If the minimum utility $U_{k}^{\min}$ is not achieved, player *k* does not cooperate. 

In non-cooperative games, each player gets the minimum utility without collaboration [26]. Assuming that $\left\{s_{1}^{*},\ldots ,s_{n}^{*}\right\}$ is the strategy set composed of a strategy for each player, if $U_{i}\left(s_{1}^{*},\ldots ,s_{i-1}^{*},s_{i}^{*},s_{i+1}^{*},\ldots ,s_{n}^{*}\right)\geq U_{i}\left(s_{1}^{*},\ldots ,s_{i-1}^{*},s_{j}^{*},s_{i+1}^{*},\ldots ,s_{n}^{*}\right),\;\forall s_{j}\in S_{i},\left\{s_{1}^{*},\ldots ,s_{n}^{*}\right\}$ is the optimal stable solution, which is called the Nash equilibrium (NE). The NE is a fixed point; thus, no player can improve its utility by changing its strategy unilaterally [26]. Because non-cooperative games emphasize the individual rationality and optimal decisions for individuals, the NE is not efficient all the time [32].

Cooperative games focus more on the collaboration between players to pursue the most advantageous strategy, which is usually collectively optimal. Let $\Gamma =\left\{U_{1},\ldots ,U_{n}\right\}\subset {ℜ}^{n}$ represent the feasible utility-allocation set if all the players cooperate, which is convex and closed; $\left\{U_{k}\in \Gamma |U_{k}\geq U_{k}^{\min},\forall k\in K\right\}$ is a non-empty and bounded set, and $d=\left\{U_{1}^{\min},\ldots ,U_{k}^{\min}\right\}$ is the disagreement point. The pair {Γ,*d*} constructs a K-player bargaining problem. We define the Pareto-optimal point among multiple players. It is impossible to find another point that improves the utility for all the players simultaneously [26].

**Definition** **1:** *(Pareto optimality): The resource-allocation point Γ is Pareto-optimal if and only if any allocation $U_{k}^{\prime }$* *satisfies* $U_{k}^{\prime }\geq U_{k},\forall k\in K$ *when* $U_{k}^{\prime }=U_{k}.$

For multiple players, there may be an infinite number of Pareto-optimal points [32]. Thus, we must select the best Pareto point, which requires a criterion for the bargaining solution. Multiple bargaining selection criteria can be used for our energy-efficiency problem (e.g., they consider optimality and fairness) [32]. We investigate a well-known Nash bargaining solution (NBS), which can provide a fair and unique Pareto optimal point satisfying the following axioms.

**Definition** **2:** *Let F be a function* $F:\left(\Gamma ,d\right)\to {ℜ}^{n}$*). Let B be the set of Pareto-optimal points, i.e., all the individually rational utility points in the cooperative utility region.* $U^{*}=F\left(\Gamma ,d\right)$ *is said to be an NBS in Γ for d if the following axioms are satisfied* [26]*:*

*(1)*  *Individual rationality:* $U_{k}^{*}\geq d_{k}$ *for* $U_{k}^{*}\in U^{*},\forall k\in Κ$*.*
*(2)*  *Feasibility:*$\;U^{*}\in \Gamma$ *.*
*(3)*  Pareto-optimality: U* is Pareto-optimal.
*(4)*  *Independence of irrelevant alternatives: if* $U^{*}\in \Gamma^{\prime }\subset \Gamma$ *and* $U^{*}=F\left(\Gamma ,d\right)$*, then* $U^{*}=F\left(\Gamma^{\prime },d\right)$ *.*
*(5)*  *Independence of linear transformations: For any linear scale transformation* ψ, ψ(F(Γ,d))=F(ψ(Γ),ψ(d)).
*(6)*  *Symmetry: if Γ is invariant under all exchanges of players (base stations),* $F_{i}\left(\Gamma ,d\right)=F_{j}\left(\Gamma ,d\right),\forall i,j$*.*

Axioms (1), (2), and (3) define the bargaining set B. Hence, the NBS is located in the bargaining set. Axioms (4), (5), and (6) are called fairness axioms. Axiom (4) shows that if the bargaining solution of the larger set exists in a smaller domain, the solution is not affected by expanding the domain, which ensures that the NBS is invariant by limiting the maximum attainable utility. Axiom (5) guarantees that the bargaining solution is invariant, which can yield the NBS in a linearly transformed domain. The symmetry Axiom (6) implies that if players have the same utility functions and disagreement points, they have the same utility. This represents a significant fairness criterion for our cooperative game that gives incentives to multiple base stations to collaborate, as they can depend on the network to provide them fair treatment when their utility resource tradeoffs vary over time.

### 3.2. Utility Design and Bargaining Cooperative Game Formulation

In our case, as energy giants of the network, the MeNBs and SeNBs are the players, which cooperate or bargain to divide the available network energy. Thus, bargaining cooperative game-based interference-aware power coordination is formulated to improve network EE. The following theorem shows the existence and uniqueness of the NBS that satisfies the aforementioned axioms.

**Theorem** **1:** *The bargaining cooperative game is: *

(15) $$G=\langle \left\{MeNB_{m},SeNB_{n},m\in M,n\in Ν\right\};\left\{p_{m},p_{n},m\in M,n\in Ν\right\};u\rangle$$

*For the players (MeNBs and SeNBs),* m∈M, n∈N *, the available actions are adjusting the transmission power* $p_{m}\leq p_{m}^{max},p_{n}\leq p_{n}^{max}.$ *Here, we introduce the Nash-product function* $u=\prod_{m=1}^{M}u_{m}\prod_{n=1}^{N}u_{n},$ *where u_m_ and u_n_ are the utility functions for MeNB_m_ and SeNB_n_, respectively. Assuming a unique and fair solution,* $F\left(\Gamma ,d\right)\in \arg\underset{u\in \Gamma ,u\geq d}{\max}\prod_{m=1}^{M}\left(u_{m}-u_{m}^{min}\right)\prod_{n=1}^{N}\left(u_{n}-u_{n}^{min}\right)$ *satisfies all the axioms in Definition 2.*

**Proof:**  The proof of this theorem is omitted because of space limitations. A similar detailed proof can be found in [33], which is proven to be efficient and fair.☐

**Remark** **1:** *It is important to design the utility function of such a game, which must reflect the influence of the changing strategies of the base stations on the network EE. In previous studies, game theory-based power coordination for SE and EE optimization, which achieves an optimal tradeoff between SE and EE, was widely adopted as the utility function. However, the DE, which is another critical issue for measuring the network EE when multiple SeNBs are densely overlaid on the macro-cell, has been neglected in these game-theoretic formulations.*

In order to jointly address SE, DE, and EE, and obtain an optimal tradeoff between them, *u_m_* and *u_n_* are introduced with two adjustable parameters *α* and *β*.

**Lemma** **1:** *The utility function u_m_ of a player MeNB_m_, m* *∈ M, is defined as* $u_{m}={\left(EE_{m}-EE_{m}^{0}\right)}^{\alpha_{m}}{\left(DE_{m}-DE_{m}^{0}\right)}^{\beta_{m}}{\left(SE_{m}-SE_{m}^{0}\right)}^{1-\alpha_{m}-\beta_{m}}.$ *The utility function u_n_ of a player SeNB_n_, n ∈ N, is defined as* $u_{n}={\left(EE_{n}-EE_{n}^{0}\right)}^{\alpha_{n}}{\left(DE_{n}-DE_{n}^{0}\right)}^{\beta_{n}}{\left(SE_{n}-SE_{n}^{0}\right)}^{1-\alpha_{n}-\beta_{n}},$ *where* $EE_{m}$*,* $DE_{m}$*,* $SE_{m}$*,* $EE_{n}$*,* $DE_{n}$*, and* $SE_{n}$ *are the EE, DE, and SE functions, which can be calculated using Equations (9)–(15).* $EE_{m}^{0}$*,* $DE_{m}^{0}$ *and* $SE_{m}^{0}$ *represent the minimum requirements of the EE, DE, and SE, respectively, toward the MeNB_m_.* $EE_{n}^{0}$*,* $DE_{n}^{0}$ *and* $SE_{n}^{0}$ *represent the minimum requirements of the EE, DE, and SE, respectively, toward the SeNB_n_. The coefficients 0 ≤ α_m_ ≤ 1, 0 ≤ β_m_ ≤ 1, 0 ≤ α_n_ ≤ 1, and 0 ≤ β_n_ ≤ 1 are adopted to maintain a balance between the EE, DE, and SE.*

**Remark** **2:** *In contrast to the utility function in [31], our utility function is more comprehensive and considers the cost. It reveals the effect of the power-coordination behaviors between different base stations on the network cost. On the other hand, the coefficients α_m_,* *β_m_, α_n_ and* *β_n_ are offered for players to balance the three energy-efficiency metrics. They are determined by the distribution of users associated with SeNB_n_ and MeNB_m_. If a base station is associated with a low number of users, the downlink power is coordinated to focus more on the EE and DE; otherwise, the SE is considered. If α_m_ = 1 and* *β_m_ = 0, MeNB_m_ only considers the EE, and if α_m_ = 0 and* *β_m_ = 1, MeNB_m_ only considers the DE. If α_m_ = 0 and* *β_m_ = 0, MeNB_m_ only considers the spectrum efficiency.*

Therefore, the unique Nash bargaining game equilibrium can be achieved by solving the following Nash optimization problem. By adopting the objective utility function in Lemma 1, the optimization problem can be rewritten as:

(16) $$P1:\underset{0\leq p_{m}\leq p_{m}^{MAX},0\leq p_{n}\leq p_{n}^{MAX}}{\max}\prod_{m=1}^{M}\left(u_{m}-u_{m}^{\min}\right)\prod_{n=1}^{N}\left(u_{n}-u_{n}^{\min}\right)$$

$$\text{subject to }C1:EE_{m}-EE_{m}^{0}\geq 0,m\in M,$$

$$C2:\;DE_{m}-DE_{m}^{0}\geq 0,m\in M,$$

$$C3:\;SE_{m}-SE_{m}^{0}\geq 0,m\in M,$$

$$C4:EE_{n}-EE_{n}^{0}\geq 0,n\in Ν,$$

$$C5:DE_{n}-DE_{n}^{0}\geq 0,n\in Ν,$$

(17) $$C6:SE_{n}-SE_{n}^{0}\geq 0,n\in Ν$$

## 4. Bargaining Problem Solutions for Ultradense Wireless Cellular Networks

### 4.1. Problem Simplification

The problem in Equation (16) is too complicated to be analyzed and solved. Therefore, some simplifications are made. On the basis of numerous studies focusing on the SINR-based optimal functions in [20,23], the CINR functions in Equations (3) and (5) are used instead of the achievable capacity in Equations (6) and (7) in the utility functions of Lemma 2. According to the analysis in [15], *c*_0_ is far smaller than *c*_1_, which indicates that we can omit the CapEx *c*_0_ in the utility functions of Lemma 2 for simplicity:

**Corollary** **1:** *The optimization problem of P1 in Equation (16) is simplified as:*

(18) $$P2:\underset{0\leq p_{m}\leq p_{m}^{MAX},0\leq p_{n}\leq p_{n}^{MAX}}{\max}\psi =\prod_{m=1}^{M}\frac{p_{m}h_{m}}{{\left(a_{sitem}*p_{m}+b_{sitem}\right)}^{\alpha_{m}+\beta_{m}}\cdot c_{1m}{}^{\beta_{m}}}\cdot \prod_{n=1}^{N}\frac{p_{n}h_{n}}{{\left(a_{siten}*p_{n}+b_{siten}\right)}^{\alpha_{n}+\beta_{n}}\cdot c_{1n}{}^{\beta_{n}}}$$

*where* $h_{m}=\sum_{i=1}^{I}h_{m,i}$ *and* $h_{n}=\sum_{j=1}^{I}h_{n,j}$ *are the aggregate CINRs from MeNB_m_ and SeNB_n_ to their corresponding MUEs and SUEs, respectively.*

**Proof:**  According to Equations (8)–(11), (13) and (14), the utility functions of u_m_ and u_n_ of Lemma 1 can be expressed as:

(19) $$\begin{array}{l} u_{m}={\left[\frac{\sum_{i=1}^{I}\log_{2}(1+p_{m}h_{m,i})}{a_{sitem}*p_{m}+b_{sitem}}\right]}^{\alpha_{m}}\cdot {\left[\frac{\sum_{i=1}^{I}\log_{2}(1+p_{m}h_{m,i})}{c_{1m}*(a_{sitem}*p_{m}+b_{sitem})+c_{om}}\right]}^{\beta_{m}}\cdot {\left[\sum_{i=1}^{I}\log_{2}(1+p_{m}h_{m,i})\right]}^{1-\alpha_{m}-\beta_{m}}\\ =\frac{\sum_{i=1}^{I}\log_{2}(1+p_{m}h_{m,i})}{{\left(a_{sitem}*p_{m}+b_{sitem}\right)}^{\alpha_{m}}\cdot {\left(c_{1m}*(a_{sitem}*p_{m}+b_{sitem})+c_{om}\right)}^{\beta_{m}}} \end{array}$$

(20) $$\begin{array}{l} u_{n}={\left[\frac{\sum_{j=1}^{J}\log_{2}(1+p_{n}h_{n,j})}{a_{siten}*P_{n}+b_{siten}}\right]}^{\alpha_{n}}\cdot {\left[\frac{\sum_{j=1}^{J}\log_{2}(1+p_{n}h_{n,j})}{c_{1n}*(a_{siten}*P_{n}+b_{siten})+c_{0n}}\right]}^{\beta_{n}}\cdot {\left[\sum_{j=1}^{J}\log_{2}(1+p_{n}h_{n,j})\right]}^{1-\alpha_{n}-\beta_{n}}\\ =\frac{\sum_{j=1}^{J}\log_{2}(1+p_{n}h_{n,i})}{{\left(a_{siten}*p_{n}+b_{siten}\right)}^{\alpha_{n}}\cdot {\left(c_{1n}*(a_{siten}*p_{n}+b_{siten})+c_{on}\right)}^{\beta_{n}}} \end{array}$$

As the CINR definitions in Equations (3) and (5) are used instead of the achievable capacity from Equations (6) and (7), the sum achievable capacities of MeNB_m_ and SeNB_n_ are:

(21) $$C_{m}=\sum_{i=1}^{I}p_{m}h_{m,i}=p_{m}h_{m}$$

(22) $$C_{n}=\sum_{j=1}^{J}p_{n}h_{n,j}=p_{n}h_{n}$$

where $h_{m}=\sum_{i=1}^{I}h_{m,i}$ and $h_{n}=\sum_{j=1}^{I}h_{n,j}$ are the aggregate CINRs from all the MUEs and SUEs of the corresponding MeNBs and SeNBs, respectively.

Thus, the utility functions in Equations (19) and (20) can be simplified as:

(23) $$u_{m}=\frac{p_{m}h_{m}}{{\left(a_{sitem}*p_{m}+b_{sitem}\right)}^{\alpha_{m}}\cdot {\left(c_{1m}*(a_{sitem}*p_{m}+b_{sitem})+c_{om}\right)}^{\beta_{m}}}$$

(24) $$u_{n}=\frac{p_{n}h_{n}}{{\left(a_{siten}*p_{n}+b_{siten}\right)}^{\alpha_{n}}\cdot {\left(c_{1n}*(a_{siten}*p_{n}+b_{siten})+c_{on}\right)}^{\beta_{n}}}$$

with the assumptions of $EE_{m}^{0}=DE_{m}^{0}=SE_{m}^{0}=0$ and $EE_{n}^{0}=DE_{n}^{0}=SE_{n}^{0}=0$.

In addition, according to the analysis, the CapEx c_0_ is far smaller than the OpEx coefficient c_1_; that is, the CapEx c_0m_ and c_0n_ in the utility functions of Equations (23) and (24) can be omitted compared with c_1m_*(a_sitem_*p_m_*b_sitem_) and c_1n_*(a_siten_*p_n_*b_siten_). Thus, the utility functions can be further simplified:

(25) $$u_{m}=\frac{p_{m}h_{m}}{{\left(a_{sitem}*p_{m}+b_{sitem}\right)}^{\alpha_{m}+\beta_{m}}\cdot c_{1m}{}^{\beta_{m}}}$$

(26) $$u_{n}=\frac{p_{n}h_{n}}{{\left(a_{siten}*p_{n}+b_{siten}\right)}^{\alpha_{n}+\beta_{n}}\cdot c_{1n}{}^{\beta_{n}}}$$

Therefore, the optimization problem of P2 can be easily obtained with mathematical derivations by Equations (25) and (26) in Equation (16).☐

**Corollary** **2:** *The problem P2 in Equation (18) can be equivalently converted into:*

(27) P3: max ξ

$$s.t.\;C1:0\leq p_{m}\leq p_{m}^{MAX}$$

(28) $$C2:0\leq p_{n}\leq p_{n}^{MAX}$$

*where the utility function is* ξ=ln(ψ)*.*

**Proof:**  Because the logarithmic function does not change the convexity and the utility function in Equation (18) is proven to be convex according to [33], P3 in Equation (27) is equal to P2 in Equation (18).

Then, the problem in Equation (27) can be expanded as:

(29) $$\begin{array}{l} \max\{\sum_{m=1}^{M}\left\{\ln p_{m}+\ln h_{m}-(\alpha_{m}+\beta_{m})\ln(a_{sitem}*p_{m}+b_{sitem})\right\}+\\ \sum_{n=1}^{N}\left\{\ln p_{n}+\ln h_{n}-(\alpha_{n}+\beta_{n})\ln(a_{siten}*p_{n}+b_{siten})\right\}\} \end{array}$$

According to the aforementioned analysis of the system model, the aggregate CINRs *h_m_* and *h_n_* are functions of the inter-tier interference. To reflect the influence of the inter-tier interference on the network EE, the problem (29) can be divided into the MeNB problem and the SeNB problem:

(30a) $$\text{P-MeNB}:\text{ max }\sum_{m=1}^{M}\left\{\ln p_{m}-(\alpha_{m}+\beta_{m})\ln(a_{sitem}*p_{m}+b_{sitem})\right\}+\sum_{n=1}^{N}\ln h_{n}$$

(30b) $$s.t.\;0\leq p_{m}\leq p_{m}^{MAX}$$

(31a) $$\text{P-SeNB}:\text{ max }\sum_{n=1}^{N}\left\{\ln p_{n}-(\alpha_{n}+\beta_{n})\ln(a_{siten}*p_{n}+b_{siten})\right\}+\sum_{m=1}^{M}\ln h_{m}$$

(31b) $$s.t.\;0\leq p_{n}\leq p_{n}^{MAX}$$

☐

### 4.2. Solution of Cooperative Bargaining Game

According to the aforementioned analysis, we can reformulate the problem in Equations (30) and (31). For deriving the closed-form solutions of this problem, we assume that a wired backhaul exists to connect the MeNBs to the SeNBs; that is, the interference information can be exchanged between them for cooperative power coordination. This assumption was made in [31]. The general EE-coordination problem in the presence of inter-tier and intra-tier interference is difficult to solve even under the precondition of ideal information exchange. Hence, a simplified scheme is proposed to reduce the information-exchange overhead, which solves the aforementioned problem by updating the transmission power.

**Corollary** **3:** *The closed-form power solution of P-MeNB in Equation (30) is given by:*

(32) $$p_{m}^{*}=\frac{1-\alpha_{m}-\beta_{m}}{\sum_{n=1}^{N}\sum_{j=1}^{J}\frac{g_{m,\{n,j\}}}{g_{n,j}}\frac{h_{n,j}^{2}}{h_{n}}+\lambda_{m}}$$

*where* $\lambda_{m}$ *is the Lagrange multiplier corresponding to the MeNB_m_ power constraint.*

**Proof:**  For the primal problem P3 expanded in Equation (30), introducing the Lagrangian multipliers $\lambda_{m}$ corresponding to the MeNB_m_ power constraint $0\leq p_{m}\leq p_{m}^{MAX}$, which yields:

(33) $$L_{m}=\sum_{m=1}^{M}\left\{\ln p_{m}-(\alpha_{m}+\beta_{m})\ln(a_{sitem}*p_{m}+b_{sitem})\right\}+\sum_{n=1}^{N}\ln h_{n}-\sum_{m=1}^{M}\lambda_{m}(p_{m}-p_{m}^{MAX})$$

On the basis of standard optimization techniques and the Karush–Kuhn–Tucker (KKT) conditions [26], the power allocation for MeNB_m_ is determined by obtaining the first-order derivative of Equation (33) with respect to $p_{m}$, which can be given as:

(34) $$\frac{\partial L_{m}}{\partial p_{m}}=\frac{1}{p_{m}}-\frac{(\alpha_{m}+\beta_{m})\cdot a_{sitem}}{a_{sitem}*p_{m}+b_{sitem}}+\sum_{n=1}^{N}\frac{1}{h_{n}}\frac{\partial h_{n}}{\partial p_{m}}-\lambda_{m}$$

According to the definition of $h_{n}=\sum_{j=1}^{I}h_{n,j}$ and the definition of *h_n,j_* in Equation (5), we obtain:

(35) $$\begin{array}{l} \frac{\partial h_{n}}{\partial p_{m}}=\frac{\partial }{\partial p_{m}}\left\{\sum_{j=1}^{J}h_{n,j}\right\}=\sum_{j=1}^{J}\frac{\partial h_{n,j}}{\partial p_{m}}\\ =\sum_{j=1}^{J}\frac{\partial }{\partial p_{m}}\frac{g_{n,j}}{\sum_{n\prime \neq n,n\prime =1}^{N}p_{n\prime }g_{n\prime ,\{n,j\}}+\sum_{m=1}^{M}p_{m}g_{m,\{n,j\}}+\sigma_{n,j}^{2}}\\ =-\sum_{j=1}^{J}\frac{g_{m,\{n,j\}}}{g_{n,j}}h_{n,j}^{2} \end{array}$$

By substituting Equation (35) into Equation (34), we obtain:

(36) $$\begin{array}{l} \frac{\partial L_{m}}{\partial p_{m}}=\frac{1}{p_{m}}-\frac{(\alpha_{m}+\beta_{m})\cdot a_{sitem}}{a_{sitem}*p_{m}+b_{sitem}}-\sum_{n=1}^{N}\frac{1}{h_{n}}\sum_{j=1}^{J}\frac{g_{m,\{n,j\}}}{g_{n,j}}h_{n,j}^{2}-\lambda_{m}\\ =\frac{1}{p_{m}}-\frac{(\alpha_{m}+\beta_{m})\cdot a_{sitem}}{a_{sitem}*p_{m}+b_{sitem}}-\sum_{n=1}^{N}\sum_{j=1}^{J}\frac{h_{n,j}^{2}}{h_{n}}\frac{g_{m,\{n,j\}}}{g_{n,j}}-\lambda_{m} \end{array}$$

According to the analysis in [15], the circuit power consumption $b_{sitem}$ is constant. Here, to obtain the closed-form solution of P-MeNB in Equation (30), we assume that $b_{sitem}=0$. Then,

(37) $$\begin{array}{l} \frac{\partial L_{m}}{\partial p_{m}}=\frac{1}{p_{m}}-\frac{(\alpha_{m}+\beta_{m})\cdot a_{sitem}}{a_{sitem}*p_{m}}-\sum_{n=1}^{N}\sum_{j=1}^{J}\frac{h_{n,j}^{2}}{h_{n}}\frac{g_{m,\{n,j\}}}{g_{n,j}}-\lambda_{m}\\ =\frac{1}{p_{m}}-\frac{\left(\alpha_{m}+\beta_{m}\right)}{p_{m}}-\sum_{n=1}^{N}\sum_{j=1}^{J}\frac{h_{n,j}^{2}}{h_{n}}\frac{g_{m,\{n,j\}}}{g_{n,j}}-\lambda_{m} \end{array}$$

Finally, the closed-form solution of MeNB_m_ can be obtained, as follows:

(38) $$p_{m}^{*}=\frac{1-\alpha_{m}-\beta_{m}}{\sum_{n=1}^{N}\sum_{j=1}^{J}\frac{g_{m,\{n,j\}}}{g_{n,j}}\frac{h_{n,j}^{2}}{h_{n}}+\lambda_{m}}$$

☐

**Remark** **3:** *According to Equation (38), we conclude the following:*

*(1)*  When α_m_ is large, MeNB_m_ focuses more on the EE (e.g., α_m_ > 1/3); thus, less transmission power is needed for MeNB_m_.
*(2)*  When β_m_ is large, MeNB_m_ focuses more on the DE (e.g., β_m_ > 1/3); thus, less transmission power is needed for MeNB_m_.
*(3)*  When N and J are large, that is, many SeNBs with many SUEs are deployed in the macro-cell controlled by MeNB_m_, if these SeNBs are deployed near MeNB_m_, as a result, MeNB_m_ provides a lower transmission power. In this sense, the strategy takes full account of the benefits to the opponents.
*(4)*  When h_n_ is large, the aggregate CINRs of all SUEs are good enough; thus, MeNB_m_ can provide a large transmission power to enhance its capacity.

**Corollary** **4:** *The closed-form power solution of P-SeNB in Equation (31) is given by:*

(39) $$p_{n}^{*}=\frac{1-\alpha_{n}-\beta_{n}}{\sum_{m=1}^{M}\sum_{i=1}^{I}\frac{g_{n,\{m,i\}}}{g_{m,i}}\frac{h_{m,i}^{2}}{h_{m}}+\lambda_{n}}$$

*where* $\lambda_{n}$ *is the Lagrange multiplier corresponding to the SeNBn power constraint.*

**Proof:**  Similarly, for the problem in Equation (31), introducing the Lagrangian multipliers $\lambda_{n}$ corresponding to the SeNB_n_ power constraint $0\leq p_{n}\leq p_{n}^{MAX}$, which yields:

(40) $$L_{n}=\sum_{n=1}^{N}\left\{\ln p_{n}-(\alpha_{n}+\beta_{n})\ln(a_{siten}*p_{n}+b_{siten})\right\}+\sum_{m=1}^{M}\ln h_{m}-\sum_{n=1}^{N}\lambda_{n}(p_{n}-p_{n}^{MAX})$$

According to the KKT conditions, the power allocation for SeNB_n_ is determined by obtaining the first-order derivative in Equation (40) with respect to $p_{n}$, which is given as:

(41) $$\frac{\partial L_{n}}{\partial p_{n}}=\frac{1}{p_{n}}-\frac{(\alpha_{n}+\beta_{n})\cdot a_{siten}}{a_{siten}*p_{n}+b_{siten}}+\sum_{m=1}^{M}\frac{1}{h_{m}}\frac{\partial h_{m}}{\partial p_{n}}-\lambda_{n}$$

According to the definition of $h_{m}=\sum_{i=1}^{I}h_{m,i}$ and the definition of *h_m,i_* in Equation (2), we obtain:

(42) $$\frac{\partial h_{m}}{\partial p_{n}}=\frac{\partial }{\partial p_{n}}\left\{\sum_{i=1}^{I}h_{m,i}\right\}=\sum_{i=1}^{I}\frac{\partial h_{m,i}}{\partial p_{n}}=-\sum_{i=1}^{I}\frac{g_{n,\{m,i\}}}{g_{m,i}}h_{m,i}^{2}$$

By substituting Equation (42) into Equation (41), we obtain:

(43) $$\frac{\partial L_{n}}{\partial p_{n}}=\frac{1}{p_{n}}-\frac{(\alpha_{n}+\beta_{n})\cdot a_{siten}}{a_{siten}*p_{n}+b_{siten}}-\sum_{m=1}^{M}\sum_{i=1}^{I}\frac{g_{n,\{m,i\}}}{g_{m,i}}\frac{h_{m,i}^{2}}{h_{m}}-\lambda_{n}$$

Similarly, to obtain the closed-form solution of P-SeNB, we assume that *b_siten_* = 0. Then:

(44) $$\frac{\partial L_{n}}{\partial p_{n}}=\frac{1}{p_{n}}-\frac{\left(\alpha_{n}+\beta_{n}\right)}{p_{n}}-\sum_{m=1}^{M}\sum_{i=1}^{I}\frac{g_{n,\{m,i\}}}{g_{m,i}}\frac{h_{m,i}^{2}}{h_{m}}-\lambda_{n}$$

Finally, we obtain the closed-form solution of SeNB_n_, as follows:

(45) $$p_{n}^{*}=\frac{1-\alpha_{n}-\beta_{n}}{\sum_{m=1}^{M}\sum_{i=1}^{I}\frac{g_{n,\{m,i\}}}{g_{m,i}}\frac{h_{m,i}^{2}}{h_{m}}+\lambda_{n}}$$

☐

**Remark** **4:** *According to Equation (45), we conclude the following:*

*(1)*  When α_n_ is large, SeNB_n_ focuses more on the EE (e.g., α_n_ > 1/3); thus, less transmission power is needed for SeNB_n_.
*(2)*  When β_n_ is large, SeNB_n_ focuses more on the DE (e.g., β_n_ > 1/3); thus, less transmission power is needed for SeNB_n_.
*(3)*  When M and I are large, that is, many MeNBs with many MUEs are deployed in the microcell controlled by SeNB_n_, if these MeNBs are deployed near SeNB_n_, SeNB_n_ provides a lower transmission power. In this sense, the strategy takes full account of the benefits to the opponents.
*(4)*  When h_m_ is large, the aggregate CINRs of all MUEs are good enough; thus, SeNB_n_ can provide a large transmission power to enhance its capacity.

### 4.3. Cooperative-Bargaining Power-Scheduling Algorithm

Although Equations (32) and (39) give closed-form solutions to the comprehensive energy-efficiency management problem of P3, an algorithm must be designed to provide the execution process for the equations. Therefore, Algorithm 1 is proposed as an implementation of our cooperative-bargaining power-scheduling solution, which guarantees convergence by using the subgradient method (proven in [32]).

**Algorithm 1:** Cooperative-Bargaining Power-Scheduling Algorithm1:  **Initialization:** MeNB_m_ and SeNB_n_, *m*∈M, *n*∈N, choose power levels *p_m_*(0) and *p_n_*(0); predefine the coefficients *α_m_*, *β_m_*, *α_n_*, and *β_n_*; predefine the Lagrangian parameters $\lambda_{m}^{\left(t\right)}$ and$\;\lambda_{n}^{\left(t\right)}$ at step *t*.2:  **for each** SUE of each SeNB_n_
**do**3:  gather the CINR: $h_{n,j}=\frac{\gamma_{n,j}}{p_{n}(t)}$4:  **end for**5:  So $h_{n}=\sum_{j=1}^{J}h_{n,j}$6:  **while not**
$\left|p_{m}^{\left(t+1\right)}-p_{m}^{\left(t\right)}\right|\leq \theta$ for any small θ
**do**7:  adjust MeNB_m_ ‘s power at the next step (*t* + 1) by: $p_{m}^{\left(t+1\right)}=\frac{1-\alpha_{m}-\beta_{m}}{\sum_{n=1}^{N}\sum_{j=1}^{J}\frac{g_{m,\{n,j\}}}{g_{n,j}}\frac{h_{n,j}^{2}}{h_{n}}+\lambda_{m}^{\left(t\right)}}$8:  update $\lambda_{m}^{\left(t+1\right)}$ by MeNB_m_ as: $\lambda_{m}^{\left(t+1\right)}=\lambda_{m}^{\left(t\right)}-\beta_{m}^{\left(t\right)}(p_{m}^{MAX}-p_{m}^{\left(t\right)})$9:  **end while**10: **for each** MUE of each MeNB_m_
**do**11:  gather the CINR: $h_{m,i}=\frac{\gamma_{m,i}}{p_{m}(t)}$12: **end for**13: So $h_{m}=\sum_{i=1}^{I}h_{m,i}$14: **while not**
$\left|p_{n}^{\left(t+1\right)}-p_{n}^{\left(t\right)}\right|\leq \theta$ for any small θ
**do**15:  adjust SeNB_n_ ‘s power at the next step (*t* + 1) by: $p_{n}^{\left(t+1\right)}=\frac{1-\alpha_{n}-\beta_{n}}{\sum_{m=1}^{M}\sum_{i=1}^{I}\frac{g_{n,\{m,i\}}}{g_{m,i}}\frac{h_{m,i}^{2}}{h_{m}}+\lambda_{n}^{\left(t\right)}}$16:  update $\lambda_{n}^{\left(t+1\right)}$ by SeNB_n_ as: $\lambda_{n}^{\left(t+1\right)}=\lambda_{n}^{\left(t\right)}-\beta_{n}^{\left(t\right)}(p_{n}^{MAX}-p_{n}^{\left(t\right)})$17: **end while**

The power coordination in Algorithm 1 can be implemented by the interference information exchange of all players, which is probably prohibitive for moderate-sized small-cell deployment. Thus, for ultra-dense wireless cellular networks, it is a challenge.

### 4.4. Simplified Algorithm for Sub-Optimal Solution

A simplified algorithm based on Algorithm 1 is proposed to obtain a sub-optimal power-coordination solution. As the power of the MeNBs is far larger than that of the SeNBs, we assume that only the inter-tier interference of two-tier macro-small HetNets exists. 

As denoted in Equation (1), $h_{m,i}$ is the function of the inter-tier interference, the intra-tier interference, and the noise. In Equation (2), with the increase of small cells, we assume that $\sum_{n=1}^{N}p_{n}g_{n,\left\{m,i\right\}}\gg \sum_{m^{\prime }\neq m,m\prime =1}^{M}p_{m}g_{m\prime ,\left\{m,i\right\}}+\sigma_{m,i}^{2}$; thus, it can be rewritten as:

(46) $$\gamma_{m,i}=\frac{p_{m}g_{m,i}}{\sum_{n=1}^{N}p_{n}g_{n,\{m,i\}}}=p_{m}h_{m,i}$$

that is:

(47) $$\frac{1}{h_{m,i}}=\frac{\sum_{n=1}^{N}p_{n}g_{n,\{m,i\}}}{g_{m,i}}$$

To simplify the power-updating process, we assume that there is always one maximum SeNB_n_ interference for the MUE. This assumption was made in [31]. Therefore, we have:

(48) $$\frac{1}{h_{m,i}}=\frac{p_{n}g_{n,\{m,i\}}}{g_{m,i}}$$

Then, the power update of SeNBs is:

(49) $$\begin{array}{l} p_{n}^{\left(t+1\right)}=\frac{1-\alpha_{n}-\beta_{n}}{\sum_{m=1}^{M}\sum_{i=1}^{I}\frac{g_{n,\{m,i\}}}{g_{m,i}}\frac{h_{m,i}^{2}}{h_{m}}+\lambda_{n}^{\left(t\right)}}\\ \approx \frac{1-\alpha_{n}-\beta_{n}}{\sum_{m=1}^{M}\sum_{i=1}^{I}\frac{1}{h_{m,i}p_{n}^{\left(t\right)}}\frac{h_{m,i}^{2}}{h_{m}}+\lambda_{n}^{\left(t\right)}}=\frac{1-\alpha_{n}-\beta_{n}}{\frac{1}{p_{n}^{\left(t\right)}}\sum_{m=1}^{M}\frac{1}{h_{m}}\sum_{i=1}^{I}\frac{h_{m,i}^{2}}{h_{m,i}}+\lambda_{n}^{\left(t\right)}}\\ =\frac{1-\alpha_{n}-\beta_{n}}{\frac{M}{p_{n}^{\left(t\right)}}+\lambda_{n}^{\left(t\right)}} \end{array}$$

Similarly, in Equation (4), with the increase of small cells, we assume that $\sum_{m=1}^{M}p_{m}g_{m,\left\{n,j\right\}}\gg \sum_{n^{\prime }\neq n,n\prime =1}^{N}p_{n\prime }g_{n\prime ,\left\{n,j\right\}}+\sigma_{n,j}^{2}$; thus, it can be rewritten as:

(50) $$\gamma_{n,j}=\frac{p_{n}g_{n,j}}{\sum_{m=1}^{M}p_{m}g_{m,\{n,j\}}}=p_{n}h_{n,j}$$

that is:

(51) $$\frac{1}{h_{n,j}}=\frac{\sum_{m=1}^{M}p_{m}g_{m,\{n,j\}}}{g_{n,j}}$$

In a practical ultra-dense wireless cellular network, SeNBs are deployed in the coverage of the MeNB; consequently, this MeNB interference is maximized for these overlaid SeNBs. Therefore, we have:

(52) $$\frac{1}{h_{n,j}}=\frac{p_{m}g_{m,\{n,j\}}}{g_{n,j}}$$

Then, the power update of MeNBs is:

(53) $$\begin{array}{l} p_{m}^{\left(t+1\right)}=\frac{1-\alpha_{m}-\beta_{m}}{\sum_{n=1}^{N}\sum_{j=1}^{J}\frac{g_{m,\{n,j\}}}{g_{n,j}}\frac{h_{n,j}^{2}}{h_{n}}+\lambda_{m}^{\left(t\right)}}\\ \approx \frac{1-\alpha_{m}-\beta_{m}}{\sum_{n=1}^{N}\sum_{j=1}^{J}\frac{1}{h_{n,j}p_{m}^{\left(t\right)}}\frac{h_{n,j}^{2}}{h_{n}}+\lambda_{m}^{\left(t\right)}}=\frac{1-\alpha_{m}-\beta_{m}}{\frac{1}{p_{m}^{\left(t\right)}}\sum_{n=1}^{N}\frac{1}{h_{n}}\sum_{j=1}^{J}\frac{h_{n,j}^{2}}{h_{n,j}}+\lambda_{m}^{\left(t\right)}}\\ =\frac{1-\alpha_{m}-\beta_{m}}{\frac{N}{p_{m}^{\left(t\right)}}+\lambda_{m}^{\left(t\right)}} \end{array}$$

By substituting Equations (49) and (53) into Algorithm 1, a simplified algorithm for a sub-optimal power-coordination solution is obtained.

According to Algorithm 1, the MeNB must know the interference from *N* SeNBs and relevant *J* SUEs. Likewise, each SeNB must know the interference from all *M* MeNBs and relevant *I* MUEs. Therefore, toward every MeNB and each SeNB, the interference information must be exchanged in the amounts of *O*(*N* × *J*) and *O*(*M* × *I*), respectively. In the aforementioned simplified algorithm, toward every MeNB and each SeNB, only interference information in the amount of *O*(*N*) and *O*(*M*) must be exchanged. Thus, the simplified algorithm reduces the computational complexity of Algorithm 1.

## 5. Simulation Results and Discussion 

In this section, the simulation results are shown to illustrate the convergence performance and the effectiveness of two proposed algorithms. A practical Long Term Evolution-Advanced (LTE-A) scenario is applied. In the simulations, small spectrum-sharing co-channel SeNBs and MUEs are randomly distributed in the coverage of MeNBs, and SUEs are randomly distributed in the coverage of their SeNBs. 

For comparison with the simulation in [31], we set similar simulation parameters. The MeNBs use omnidirectional antennae, which are set in the center of their coverage in a 50 m × 50 m rectangular-area model. In the macro-cell, SeNBs are overlaid in a dense deployment, in a 5 × 5 grid model. The maximum transmit power of the MeNBs is set as 46 dBm, and the number of users per macro-cell is *I* = 100. The coverage radius of the macro-cell is 500 m, and that of a small-cell is 25 m. The number of users per small-cell is *J* = 10 and the maximal transmit power of the SeNB is 20 dBm. We use the femto base station as the SeNB in our simulations. Femtocells in the 5 × 5 grid model of 3GPP-TR 36.814 are used for pass-loss models and shadowing models, and the detailed parameters are presented in Table A.2.1.1.2-3 [36]. The path-loss model from the SeNB to SUEs is *L* = 127 + 30log_10_*R* in length, and the other links are *L* = 128.1 + 37.6log_10_*R* in length, where *R* is the distance between the base station and the user in kilometers. The Gaussian noise power spectral density is *N*_0_ = −174 dBm/Hz, where the additive white Gaussian noise power is $\sigma^{2}=B/N_{0}$. The coefficients *α_m_*, *β_m_*, *α_n_*, and *β_n_* are related to the distribution of users for each base station. 

**Figure 2:**
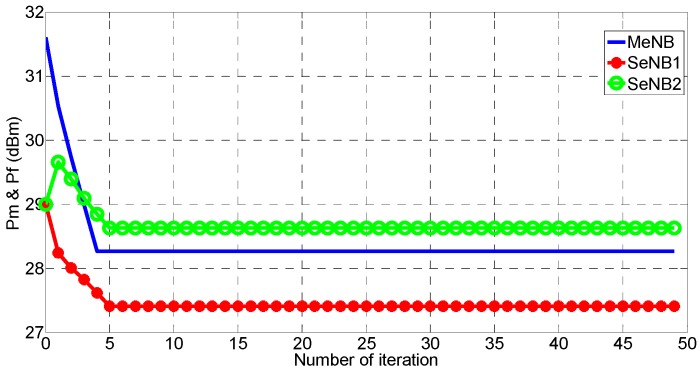
Convergence of proposed Algorithm 1.

The actual effects on the SE and EE were studied in [35]. In the present work, we set these as 1/3 to guarantee the fairness among the SE, EE, and DE. The other parameters are listed in Table 2. In Figure 2, the convergence property of Algorithm 1 is evaluated with the power iteration difference constraint θ=0.0001 and the power initializations of 46 dBm for the MeNB and 20 dBm for the other two SeNBs. This algorithm uses a fixed-point iteration process for power coordination, which is proven to converge in [24]. As shown in Figure 2, we choose the similar scenario and set the same simulation parameters as [31]. Finally, the proposed power-coordination algorithm converges in ultra-dense wireless cellular networks. As illustrated in Figure 2, *p_m_*, *p_f_*_1_ and *p_f_*_2_ exhibit fast convergence after five or six iterations. Compared with the convergence performance in [31], our algorithm ensures faster convergence.

To evaluate the performance of the proposed strategy with the increase of small cells, the area EE, area SE, and area DE of the proposed power-coordination scheme are simulated. Here, the area SE of the system, MeNB, and SeNB are calculated using the following equations, respectively, similarly to [31]:

(54) $$\text{System SE }=\frac{\sum_{n=1}^{N}SE_{n}+\sum_{m=1}^{M}SE_{m}}{3.14r^{2}}$$

(55) $$\text{MeNB SE }=\frac{\sum_{m=1}^{M}SE_{m}}{M\cdot 3.14r^{2}}$$

(56) $$\text{SeNB SE }=\frac{\sum_{n=1}^{N}SE_{n}}{N\cdot 3.14r^{2}}$$

where *r* is the radius, which we set as 500 m.

**Figure 3:**
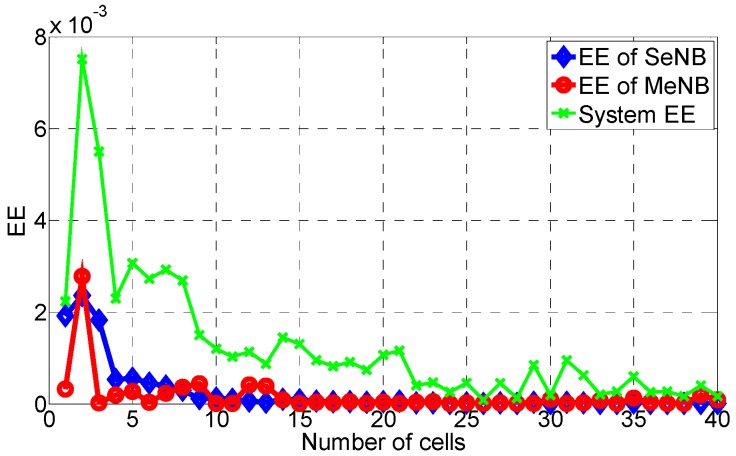
Area EE with the proposed power-coordination strategy.

**Figure 4:**
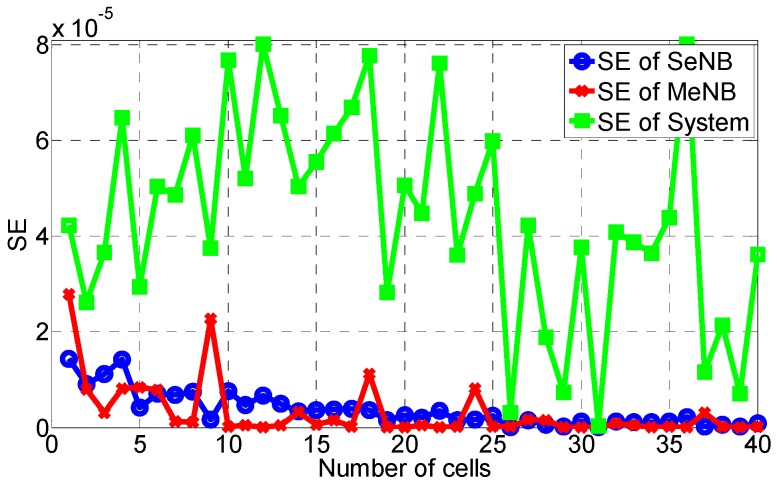
Area SE with the proposed power-coordination strategy.

**Figure 5:**
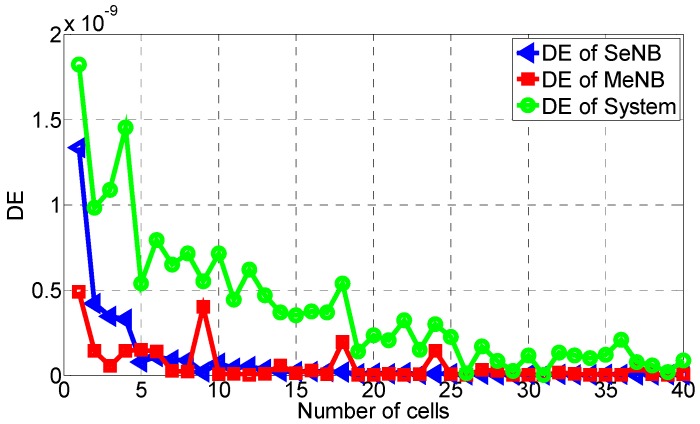
Area DE with the proposed power-coordination strategy.

Then, the area EE and DE are calculated similarly. As shown in Figure 3, Figure 4 and Figure 5, these area metrics are simulated when the number of small cells increases from 1 to 40. According to Figure 3, we conclude that the trend of the area EE of the SeNB, the MeNB and the whole system is to decrease with the increase of small cells, especially from two small cells. This is because when more small cells are overlaid in the coverage of the MeNB, the energy consumption is higher, and the interference between them increases. The same trend is observed for the area DE of the SeNB, the MeNB and the whole system, as shown in Figure 5, which is caused by the increase of the cost with the increase of small cells. However, as shown in Figure 4, the area SE of SeNB and MeNB decreases with respect to the number of small cells, but the system SE fluctuates widely. More small cells yield a larger system capacity, whereas more small cells may aggravate the interference of the whole system. Therefore, with the increase of small cells, the system performance is not always improved. That is, in network construction, there should be a good tradeoff considering the SE, EE, and DE.

**Figure 6:**
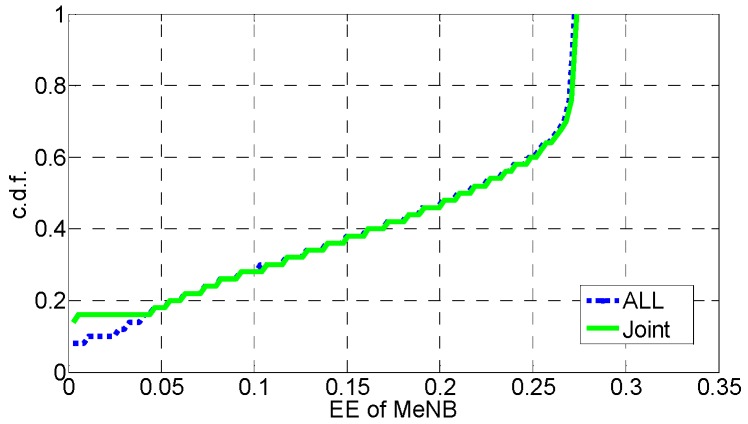
CDF of EE for MeNB.

**Figure 7:**
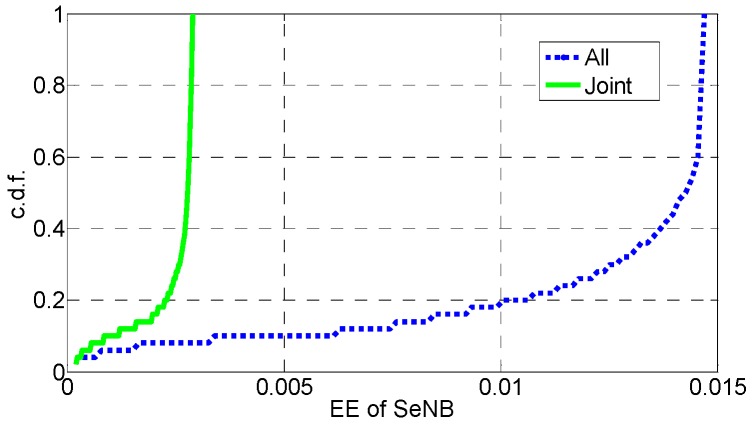
CDF of EE for SeNB.

**Figure 8:**
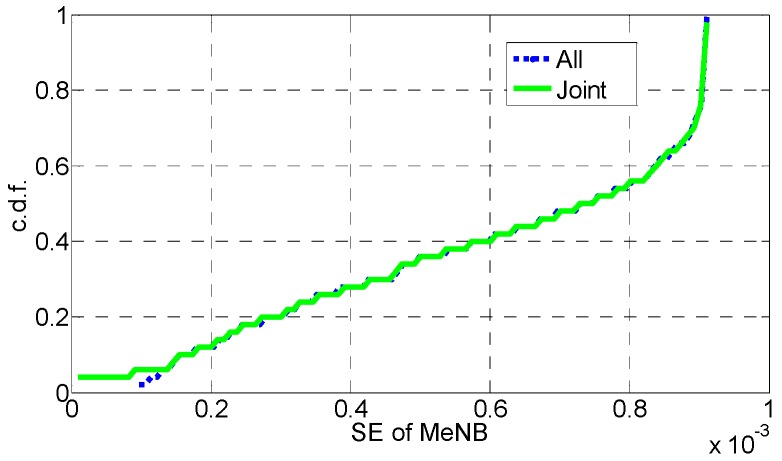
CDF of SE for MeNB.

**Figure 9:**
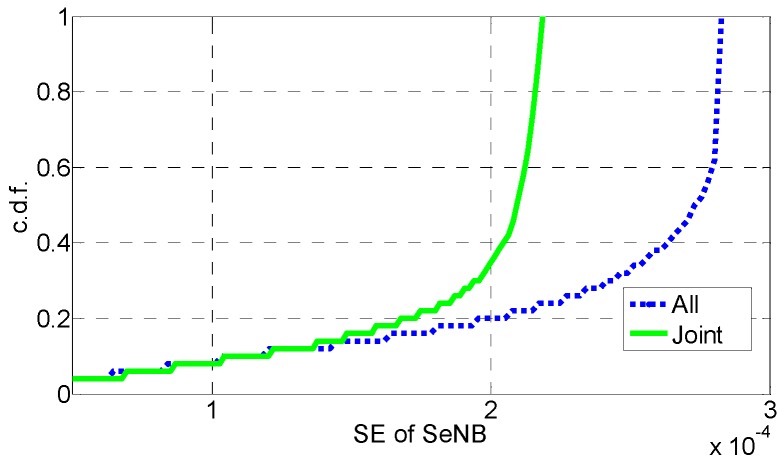
CDF of SE for SeNB.

**Figure 10:**
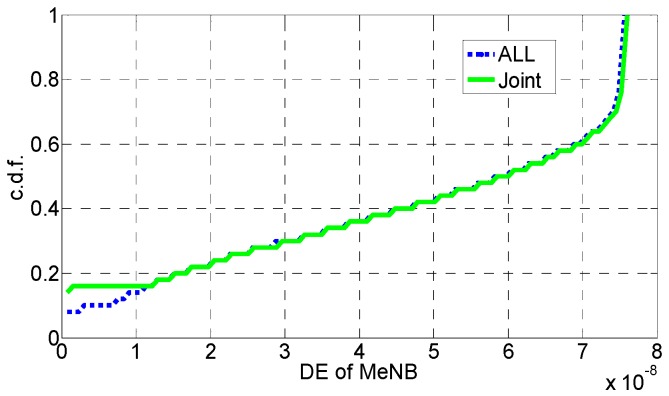
CDF of DE for MeNB.

**Figure 11:**
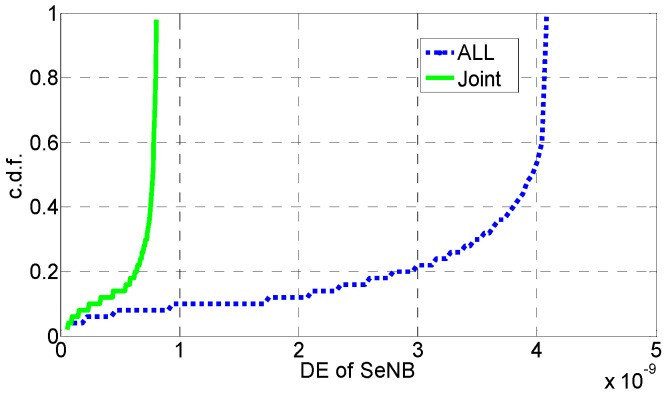
CDF of DE for SeNB.

In Figure 6, Figure 7, Figure 8, Figure 9, Figure 10 and Figure 11, the EE, SE, and DE cumulative distribution functions (CDFs) of the MeNB and the averaged EE, SE, and DE CDFs of the SeNB are simulated to reflect the performance advantages of our scheme. We also contrast them with the corresponding EE, SE, and DE CDFs of the joint scheme in [31].

**Figure 12:**
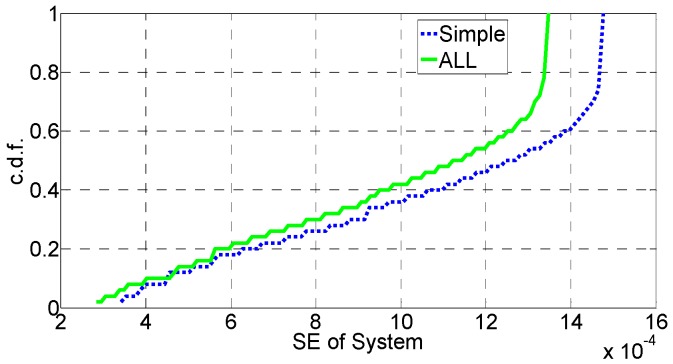
SE performance of the simplified algorithm.

**Figure 13:**
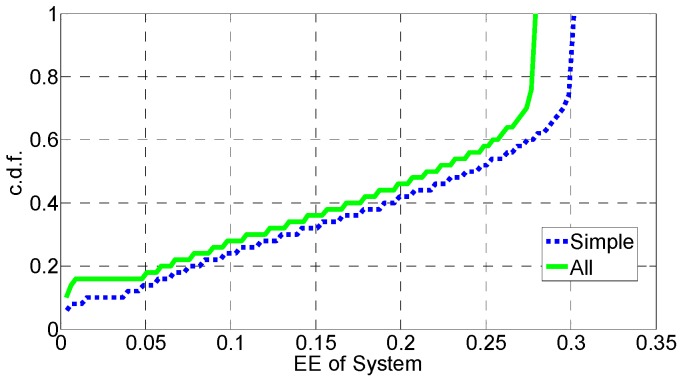
EE performance of the simplified algorithm.

**Figure 14:**
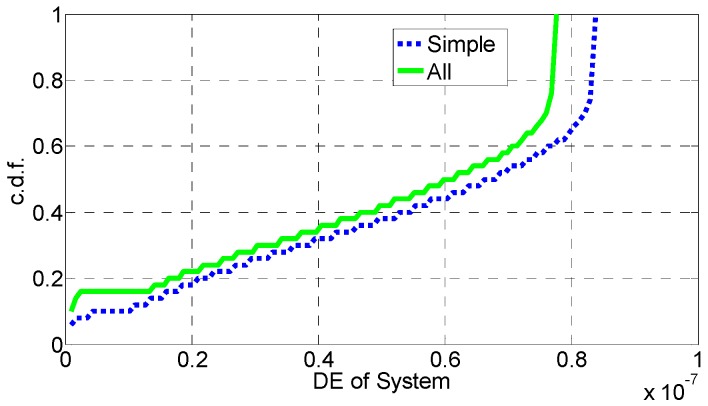
DE performance of the simplified algorithm.

According to Figure 6 and Figure 7, the EE of the MeNB is improved in our strategy; however, the averaged EE of the SeNB can be further improved. The SE and DE are also improved, as shown in Figure 9, Figure 10, Figure 11, Figure 12, Figure 13 and Figure 14. Owing to the consideration of the DE, the power of the MeNBs and SeNBs in our algorithm is coordinated slightly. Because more MeNBs are introduced, the power of the SeNBs is lower. Moreover, the CINRs of the MUEs and SUEs are smaller; therefore, the power adjustments of the MeNBs are not far better than those of the SeNBs. On the other hand, lower transmission power causes less SE but is beneficial for reducing the consumed energy and the cost, which leads to improvements in the EE and DE. Thus, we conclude that a slight sacrifice in the SE for MeNBs yields large savings in energy and cost. SeNBs obtain the biggest beneficiary of the power coordination between different base stations.

Figure 12, Figure 13 and Figure 14 illustrate the performance of our proposed simplified algorithm compared with that of Algorithm 1, which is measured via the EE, SE, and DE CDFs. As shown in these figures, the EE, SE, and DE CDFs of the simplified algorithm are close to those of Algorithm 1. Therefore, we conclude that the simplified scheme can reduce the amount of information exchanged without obvious performance degradation, which greatly decreases the complexity of the power-coordination strategy to achieve a better system EE.

## 6. Conclusions

We investigated the comprehensive EE, SE, and DE problem in a ultradense wireless cellular network. Two coefficients, *α* and *β*, were used for an adjustable utility function in a cooperative Nash bargaining game, whereby the EE problem was transformed into a power-coordination problem. Closed-form optimal interference-aware power-coordination solutions were derived by relaxing the CapEx and the circuit power consumption variables. A simplified algorithm was proposed for reducing the complexity of the signaling overhead. Accordingly, a cooperative Nash bargaining power-coordination algorithm exhibited convergence to a Pareto-optimal equilibrium for the cooperative game. According to the simulation results, the proposed power-coordination scheme not only converges within several iterations but also achieves a better tradeoff among the EE, SE, and DE. The simplified algorithm can approach the performance of the power-coordination scheme, which proves the effectiveness of this algorithm.

## Figures and Tables

**Table 1:** Key cost drivers for radio access networks.

CapEx ($C^{C_{a}}$)	OpEx ($C^{O_{p}}$)
Base-station equipment ($C_{BS}^{Ca}$)	Electric power ($C_{Power}^{Op}$)
Base-station installation and buildout ($C_{Site}^{Ca}$)	Operation and maintenance ($C_{O\&M}^{Op}$)
Backhaul transmission equipment ($C_{Trans}^{Ca}$)	Site lease ($C_{Site}^{Op}$)
Radio network controller equipment ($C_{RNC}^{Ca}$)	Backhaul transmission lease ($C_{Trans}^{Op}$)

**Table 2:** This is a table. Network simulation parameters.

Simulation Parameter	Value
Deployment scenario	5 × 5 grid model
Carrier frequency	2 GHz
Bandwidth	10 MHz
Coverage radius of the MBS	500 m
Shadowing standard deviation*2	10 dB for link between SeNB and SUE 8 dB for other links
Minimum distance between SeNB and eNB	75 m
Minimum distance between UE and eNB	35 m
Minimum distance between UE and SeNB	3 m
Minimum distance among SeNBs	40-m cluster radius
